# Consumption of ready-to-eat cereals (RTEC) among Malaysian children and association with socio-demographics and nutrient intakes – findings from the MyBreakfast study

**DOI:** 10.1080/16546628.2017.1304692

**Published:** 2017-03-27

**Authors:** Mohd Taib Mohd Nasir, Abdul Razak Nurliyana, A. Karim Norimah, Hamid Jan B. Jan Mohamed, Sue Yee Tan, Mahenderan Appukutty, Sinead Hopkins, Frank Thielecke, Moi Kim Ong, Celia Ning, E. Siong Tee

**Affiliations:** ^a^ Department of Nutrition and Dietetics, Faculty of Medicine and Health Sciences, Universiti Putra Malaysia, Serdang, Selangor, Malaysia; ^b^ Nutritional Science Programme, School of Healthcare Sciences, Faculty of Health Sciences, Universiti Kebangsaan Malaysia, Kuala Lumpur, Malaysia; ^c^ Nutrition Programme, School of Health Sciences, Universiti Sains Malaysia, Kubang Kerian, Kelantan, Malaysia; ^d^ Department of Nutrition and Dietetics, School of Health Sciences, International Medical University, Malaysia, Kuala Lumpur; ^e^ Sports Science Programme, Faculty of Sports Science and Recreation, Universiti Teknologi MARA, Shah Alam, Selangor, Malaysia; ^f^ Cereal Partners Worldwide, Lausanne, Switzerland; ^g^ T2 Goodness Ltd, Allschwil, Switzerland; ^h^ Nestlé R&D Center, Singapore; ^i^ Nutrition Society of Malaysia, c/o Division of Human Nutrition, Institute for Medical Research, Kuala Lumpur, Malaysia

**Keywords:** Breakfast foods, ready-to-eat cereals, nutrient intakes, children, Malaysia

## Abstract

**Background**: The association between different types of breakfast meals and nutrient intakes has been studied to a lesser extent.

**Objective**: This study compared nutrient intakes at breakfast and throughout the day between Malaysian children who consumed ready-to-eat cereals (RTEC) and those who did not.

**Methods**: Anthropometric and dietary data for 1955 children aged 6–12 years from the MyBreakfast study were used in the analysis.

**Results**: Overall, 18% of the children consumed RTEC at breakfast on at least one of the recall days. RTEC consumption was associated with younger age, urban areas, higher income and education level of parents. Among consumers, RTEC contributed 10% and 15% to daily intakes of calcium and iron respectively and ≥20% to daily intakes of vitamin C, thiamin, riboflavin and niacin. RTEC consumers had significantly higher mean intakes of vitamin C, thiamin, riboflavin, niacin, calcium, iron and sugar but lower intakes of fat and sodium than non-RTEC consumers at breakfast and for the total day.

**Conclusion**: Consumption of fortified RTEC at breakfast was associated with lower fat and sodium intakes and higher intakes of several micronutrients both at breakfast and for the total day. However, total sugar intakes appeared to be higher.

## Introduction

Breakfast consumption among children has been associated with higher nutritional adequacy, improved cognitive and academic performance, higher school attendance, and better mood and psychosocial functions compared to breakfast skipping.[1] Furthermore, breakfast consumption has been associated with a lower risk of excess adiposity in both children and adolescents.[2] Children who consume breakfast regularly tend to have higher intakes of total daily energy and key nutrients such as fibre, calcium, vitamins A and C, riboflavin, zinc and iron compared to those who skip breakfast.[1] The nutritional impact of different types of breakfast meals which vary in food and nutrient composition has been studied less than the breakfast occasion . Ready-to-eat cereals (RTEC) are defined as processed cereals that can be eaten without further preparation, and are also known as cold cereals. RTEC can be made of different types of grains including corn, wheat, oats and rice. Oats which need to be cooked before consumption are not considered RTEC. Cornflakes and muesli are the most common types of RTEC that are available in the Malaysian market for the general public.[3] They are generally high in carbohydrate and low in fat. Some RTEC are high in fibre and many are fortified with vitamins and minerals including B vitamins, iron and calcium. Several studies worldwide (in the USA, Europe and Australia) have shown that consumption of RTEC among children and adolescents is associated with higher daily intakes of energy from carbohydrate, total sugars, dietary fibre and several micro-nutrients (vitamins A and D, thaimin, riboflavin, niacin, vitamin B6, folate, calcium, iron, magnesium and zinc) and with a lower intake of energy from fat compared to non-consumption.[4] Moreover, consumption of RTEC has also been associated with a greater likelihood of having vitamin and mineral intakes above recommended daily requirements, especially for calcium.[4]

In Malaysia, the traditional breakfast generally includes a rice- or noodle-based hot dish such as fried rice, fried noodles and *nasi lemak* (rice cooked in coconut milk). A previous study among preschoolers aged 4–6 years in Peninsular Malaysia reported that the most common breakfast foods were bread, rice, noodles, milk and malted beverages.[5] Bread, rice dishes, noodle dishes, sweet and fried traditional cakes and biscuits were found to be the most commonly consumed foods at breakfast among adolescents in Kelantan, Malaysia,[6] while in a rural area in Tuaran, Sabah, the most common breakfast foods among adolescents were fried noodles, fried banana and doughnuts.[7]

RTEC have been available in the Malaysian market since the 1960s yet little is known about the consumption patterns of RTEC at a nationwide level and their contributions to children’s daily nutrient intake. Most RTEC are fortified with vitamins and minerals, which can be important contributors to overall nutrient intake of children. The aim of this study, therefore, was to describe the prevalence of RTEC consumption at breakfast in Malaysian children aged 6–12 years according to key socio-demographic characteristics and to compare foods consumed at breakfast and nutrient intake both at breakfast and for the total day between RTEC consumers and non-RTEC consumers.

## Materials and methods

### Study design and participants

Data of 1955 primary school children aged 6–12 years who participated in the MyBreakfast study were used in the present analysis. The MyBreakfast study was a cross-sectional study on breakfast habits of school children in Malaysia. Participants were selected using a multistage sampling method based on geographical location and ethnic group distribution. The sample size was calculated based on the total population of children aged 6–12 years in Malaysia derived from the Population and Housing Census 2010.[8] From this statistic, the percentages of children in the total population in the five regions of Malaysia, including Central, Southern, Northern, East Coast and East Malaysia, were calculated. Then for each of the regions, the percentages of children in urban and rural areas were determined. The proportion of the main ethnic ethnic groups, namely Malay, Chinese, Indian and Sabah/Sarawak native, in the urban and rural areas of each of the regions were determined. Based on this proportion, a standardized ratio of 1:1 for sex was used to determine the number of boys and girls required for each ethnic groups in the urban and rural area. The details of the sampling procedures are described elsewhere.[9,10] Data collection was carried out in the aforementioned five regions of Malaysia. A total of 33 urban and 12 rural primary schools were randomly selected based on a list of public primary schools in each of the states in Malaysia as of 31 January 2011. For each school, 2–3 classes of Primary 1 to Primary 5 were randomly selected. Children in Primary 6 were not selected in the study because these children were candidates for a national level examination and permission was not granted for these children to involve in the study. Only children who were apparently healthy and with no physical disabilities or learning difficulties were eligible to participate in this study.

Permission to conduct the study was obtained from the Ministry of Education Malaysia and Departments of Education of all the states involved. Ethical approval was obtained from Universiti Kebangsaan Malaysia Research Ethics Committee (UKMREC) and the study was conducted in accordance with the Declaration of Helsinki. A study information sheet was sent to parents together with a standard consent form provided by the UKMREC and a written consent was obtained from the parents. Data collection was conducted from April 2013 to October 2013.

### Socio-demographic characteristics

Data on socio-demographic characteristics including the child’s date of birth, sex, ethnicity, parents’education level and monthly household income were obtained from parents through a self-administered questionnaire.

### Anthropometric measurements

Height and weight of the children were measured in schools by trained researchers using standard procedures. Children were asked to empty their pockets and to take off their shoes for the measurements. Height was measured using a SECA stadiometer (SECA 217, Hamburg, Germany) to the nearest 0.1 cm, while weight was measured using a SECA digital weighing scale (SECA Clara 803, Hamburg, Germany) to the nearest 0.1 kg. All measurements were taken twice and for each measurement, the mean value was used in the analyses. Body mass index (BMI) was calculated by using the formula BMI = weight (kg)/height^2^ (m). Z-scores for BMI-for-age were determined using WHO AnthroPlus Version 1.0.3 software [11] and categorized using WHO Growth Reference 2007.[12] The cut-off points for thinness and severe thinness were −2SD and −3SD respectively, while the cut-off points for overweight and obesity were +1SD and +2SD respectively.[12]

### Dietary intake

Food and beverage intakes were assessed on one weekday and one weekend day using a food record for children aged 6–9 years and 24-h dietary recall for respondents aged 10–12 years. Parents completed the food record for children aged 6–9 years with the assistance of an instruction booklet. A brief instruction was also sent to the parents through a text message. Parents were requested to record all foods and beverages consumed by their child over the day and to note the time of consumption. The instruction booklet outlined common household measures to assist parents in the estimation of portion sizes consumed. A food album with pictures of common foods was also provided. Upon return of the dietary records in school, any incomplete food records were sent back to the parents with a short note on the uncompleted part along with a text message.

Meanwhile, a two-day 24-h dietary recall was conducted for children aged 10–12 years in a one-to-one interview by trained researchers in school. Children were requested to recall all the foods and beverages consumed in the past 24 h. Household items such as bowls, dishes, spoons and glasses in commonly used sizes, food models and a food album with pictures of common foods were used to facilitate estimation of portion sizes. For the dietary record and recall, brand information and cooking methods were also recorded where applicable.

Breakfast was defined as the first eating occasion after an overnight sleep until 10 am in the weekdays and 11 am in the weekends. Children who consumed breakfast on at least one of the two recall days were categorized as breakfast eaters, and only these children were included in the analysis. Children who consumed RTEC at breakfast on at least one of the two days were classified as RTEC consumers. Those who did not consume any RTEC at any of the two recall days were categorized as non-RTEC consumers. The mean intake of RTEC for the two recall days were used in the analysis. Mean daily energy and nutrient intakes were analysed using NutritionistPro^TM^ Software (Axxya Systems LLC, Redmond, WA, USA) based principally on the Malaysian food composition tables.[13] Sugar intake was estimated using manufacturers’ labelling as the Malaysian food composition tables do not have information on sugar in its database. Therefore for all non-packaged foods (for example, tea/coffee with condensed milk) including recipes (for example, fried rice and *nasi lemak*) there was a 0 value for total sugar assigned. Dietary data were also compared to the recommended nutrient intakes (RNI) for Malaysia.[14] An arbitrary cut-off point of 80% RNI were used to compare the adequacy of the nutrient intake of the children.

Under- and over-reporters of energy intake were determined by the ratio of mean energy intake against basal metabolic rate (BMR). Estimates of BMR for different age groups of participants were calculated using formulas from FAO/WHO/UNU [15] and predictive equations for Malaysian adolescents.[16,17] Under- and over-reporters were identified by using relevant cut-off points.[18] For boys, an under-reporter was defined as having energy intake of less than 1.39 BMR while for girls, an under-reporter was defined as having an energy intake of less than 1.30 BMR. An over-reporter was defined as having energy intake of more than 2.24 BMR for boys and more than 2.10 BMR for girls.[18]

### Statistical analyses

Data were analysed using SPSS software version 19 (IBM Corp., Armonk, NY, USA). The distribution for all variables were checked for normality and non-normally distributed variables were normalized by replacing extreme cases with group mean values. All univariate analyses were conducted using descriptive statistics. The associations between categorical variables and breakfast choice groups were determined using chi-square tests, while the associations between nutrient intakes and breakfast choice groups were determined using analysis of covariates (ANCOVA), controlling for potential confounders including indicators of socio-economic status (SES) and energy intake. The Bonferroni post hoc test was used to determine the differences between groups. The association between the type of breakfast foods consumed and recommended nutrient intake (RNI) achievement by sex and age groups was determined using chi-square tests. Level of significance was determined at *p *< 0.05.

## Results

A total of 5532 primary school children aged 6–12 years participated in the study, of which 5302 completed two days of 24-h dietary recall. It was estimated that 2648 (52.3%) of the children under-reported while 421 (8.3%) over-reported their energy intake. 1993 (39.4%) were acceptable reporters, among whom 1955 were classified as breakfast consumers (i.e. consumed breakfast on at least one of the two study days) and only these children were included in the analysis of the present paper. Table 1 shows the characteristics of acceptable and unacceptable reporters (under- and over-reporters) who were breakfast consumers. The percentage of acceptable reporters from urban (40.0%) and rural (39.5%) area was about the same. There was no difference in their family income, and father’s and mother’s educational level (*p *> 0.05). However, there were more boys who were unacceptable reporters (63.5%) compared to girls (57.5%). More children in the age group of 10–12 years were unacceptable reporters (63.3%) than children in the age group of 6–9 years (57.8%). There were also more non-RTEC consumers who were unaccaptable reporters (60.8%) than RTEC consumers (57.0%).

**Table 1.** Characteristics differences between acceptable reporters (*n *= 1955) and unacceptable reporters (*n *= 2955) who were breakfast consumers.

Characteristics	Acceptable reporters	Unacceptable reporters	*χ^2^*	*p*-value
	*n* (%)	*n* (%)		
Area			0.72	0.732
Urban	1330 (40.0)	1996 (60.0)		
Rural	625 (39.5)	959 (60.5)		
Sex			18.46	0.000
Boys	809 (36.5)	1407 (63.5)		
Girls	1146 (42.5)	1548 (57.5)		
Age groups			15.29	0.000
6 – 9 years	1171 (42.2)	1603 (57.8)		
10 – 12 years	784 (36.7)	1352 (63.3)		
Ethnic groups			30.17	0.000
Malay	1334 (41.9)	1847 (58.1)		
Chinese	263 (32.1)	557 (67.9)		
Indian	130 (35.6)	235 (64.4)		
Bumiputera Sabah/Sarawak	228 (41.9)	316 (58.1)		
Income group (MYR)			4.00	0.136
Low (<1500)	629 (40.9)	908 (59.1)		
Middle (1501–7500)	1058 (38.7)	1676 (61.3)		
High (>7500)	217 (42.7)	291 (57.3)		
Father’s education level			1.87	0.172
Secondary and below	1155 (39.1)	1797 (60.9)		
Tertiary	701 (41.2)	1002 (58.8)		
Mother’s education level			0.53	0.477
Secondary and below	1196 (39.4)	1836 (60.6)		
Tertiary	691 (40.5)	1014 (59.5)		
RTEC consumption			4.08	0.045
RTEC consumers	347 (43.0)	460 (57.0)		
Non-RTEC consumers	1608 (39.2)	2496 (60.8)		

Among the children, 17.7% consumed an RTEC at breakfast. Almost all of the children (99.9%) consumed flaked or puffed types of RTEC. All of these RTEC were fortified with B vitamins and iron, and some of them with calcium, vitamin A and vitamin C as well. The mean intake of RTEC was 23.8 g/day (*SD *= 20.1) for the total sample of cosumers. Among 6–9 year olds, the mean intake of RTEC was 21.3 g/day (*SD *= 14.9), while among 10–12 year olds, the mean intake was 28.7 g/day (*SD *= 27.2). A greater percentage of RTEC consumers were from urban areas (73.8%) compared to non-consumers (66.8%) (*p *= 0.014). There was a greater proportion of Indian children consuming a RTEC breakfast (13.0%) than non-RTEC (5.3%) breakfast (*p *< 0.001). Among RTEC consumers, there were more children in the younger age group (6–9 years) (*p *= 0.006), from higher income groups, and from families with parents who had tertiary level education compared to other breakfast consumers (*p *< 0.001). There was no significant difference in bodyweight status among RTEC consumers and non-RTEC consumers (Table 2).

**Table 2.** Socio-demographic characteristics of RTEC and non-RTEC consumers (*n *= 1955).

Variable	Total, *n*	Breakfast choice groups		*χ*^2^	*p*-value
		RTEC consumers	Non-RTEC consumers		
		*n* (%)	*n* (%)		
Overall	1955	347 (17.7)	1608 (82.3)		
Regions				16.68	0.002
Central	348	69 (19.9)	279 (17.4)		
Southern	343	83 (23.9)	260 (16.2)		
Northern	541	88 (25.4)	453 (28.2)		
East Coast	368	50 (14.4)	318 (19.8)		
East Malaysia	355	57 (16.4)	298 (18.5)		
Area				6.08	0.014
Urban	1330	256 (73.8)	1074 (66.8)		
Rural	625	91 (26.2)	534 (33.2)		
Sex				0.73	0.394
Boys	809	136 (39.2)	673 (41.9)		
Girls	1146	211 (60.8)	935 (58.1)		
Age groups				7.49	0.006
6 – 9 years	1171	231 (66.6)	940 (58.5)		
10 – 12 years	784	116 (33.4)	668 (41.5)		
Ethnic groups				29.89	<0.001
Malay	1334	229 (66.0)	1105 (68.7)		
Chinese	263	34 (9.8)	229 (14.2)		
Indian	130	45 (13.0)	85 (5.3)		
Bumiputera Sabah/Sarawak	228	39 (11.2)	189 (11.8)		
Income groups (MYR^†^) (*n *= 1905)				17.21	<0.001
Low (<1500)	629	81 (23.8)	548 (35.0)		
Middle (1501–7500)	1058	209 (61.5)	849 (54.3)		
High (>7500)	217	50 (14.7)	167 (10.7)		
Father’s education level^‡^ (*n *= 1856)				12.13	<0.001
Secondary and below	1155	180 (53.7)	975 (64.1)		
Tertiary	701	155 (46.3)	546 (35.9)		
Mother’s education level^‡^ (*n *= 1887)				11.60	0.001
Secondary and below	1196	187 (55.2)	1009 (65.2)		
Tertiary	691	152 (44.8)	539 (34.8)		
Bodyweight status^§^				2.14	0.544
Severely thin and thin	186	39 (11.2)	147 (9.1)		
Normal	1481	253 (72.9)	1228 (76.4)		
Overweight	166	32 (9.2)	134 (8.3)		
Obese	122	23 (6.6)	99 (6.2)		

^†^MYR – Malaysian Ringgit

^‡^Secondary and below: receieved ≤12 years of formal education; Tertiary: receieved >12 years of formal education

^§^Based on BMI-for-age z-scores: Severely thin and thin (z-score<-2SD); Normal (−2SD≤z-score≤+1SD); Overweight (+1SD<z-score≤+2SD) and obese (z-score>+2SD)

Table 3 outlines the top 10 food and beverage items consumed at breakfast by RTEC consumers and non-RTEC consumers. Overall, in the total sample of Malaysian children, the most common breakfast items reported were malted beverages (64.4%), bread (40.9%), condensed milk (37.3%), sugar and preserves (34.1%), eggs (29.2%) and *nasi lemak* (21.2%). Among RTEC consumers, malted beverages, ultra-heat treated (UHT) milk, condensed milk, bread and powdered milk were the most common items reported as being consumed. Malted beverages, bread, condensed milk, sugar and preserves, eggs and *nasi lemak* were the most common breakfast items consumed by non-RTEC consumers (Table 3).

**Table 3.** List of top 10 most frequently consumed food items at breakfast by all the children, RTEC consumers and non-RTEC consumers.

All children (*n *= 1955)	%	Mean±SD^†^	RTEC consumers (*n *= 347)	%	Mean±SD^†^	Non-RTEC consumers (*n *= 1608)	%	Mean±SD^†^
Malted beverage	64.4	20.4 ± 23.3	RTEC	100.0	23.8 ± 20.1	Malted beverage	64.1	20.6 ± 24.0
Bread	40.9	40.1 ± 32.6	Malted beverage	65.7	19.7 ± 20.2	Bread	44.2	40.8 ± 33.6
Condensed milk	37.3	10.7 ± 9.1	UHT milk	56.5	117.5 ± 68.2	Condensed milk	36.7	10.7 ± 9.3
Sugar and preserves	34.1	9.1 ± 15.3	Condensed milk	39.8	10.7 ± 8.6	Sugar and preserves	36.7	9.4 ± 16.2
Eggs	29.2	29.3 ± 23.8	Bread	25.6	34.5 ± 23.1	Eggs	31.8	29.4 ± 24.9
*Nasi lemak*^‡^	21.2	102.7 ± 47.1	Powdered milk	25.1	33.0 ± 40.9	*Nasi lemak*	23.9	102.6 ± 47.6
Tea/coffee	20.7	106.1 ± 81.1	Sugar and preserves	22.2	6.7 ± 4.4	Tea/coffee	22.4	103.6 ± 82.6
Chicken and meat	20.2	55.3 ± 38.2	Eggs	17.3	28.5 ± 10.4	Fried rice	21.6	138.4 ± 78.2
Fried rice	19.3	137.8 ± 77.5	Chicken and meat	14.7	46.1 ± 33.4	Chicken and meat	21.4	56.7 ± 38.7
RTEC	17.7	23.8 ± 20.1	Unleavened bread	14.1	58.4 ± 29.6	Processed fish	19.7	14.1 ± 16.1

^†^All measurement units are in grams (g), except for tea/coffee and UHT milk, which are in mililitres (ml)

^‡^ *Nasi lemak* – rice cooked in coconut milk.

In Table 4, nutrient intakes at breakfast and for the whole day are compared between RTEC consumers and non-RTEC consumers. With regards to macronutrient intakes, RTEC consumers had a significantly higher percentage of energy from carbohydrate than non-RTEC consumers both at breakfast (57.7% versus 55.1%; *p *< 0.001) and for the total day (50.3% versus 49.2%; *p *= 0.011). RTEC consumers also had a significantly lower energy contribution from fat at breakfast (29.6% versus 33.6%; *p *< 0.001) and this difference remained statistically signficant for daily intakes (*p *= 0.048), while there was no significant difference in protein intakes at breakfast or for the total day. Sodium intakes at breakfast (391 mg versus 574 mg) and for the total day (1901 mg versus 2038 mg) were lower in RTEC consumer than non-RTEC consumers (*p *< 0.001). RTEC consumers had higher intakes of vitamin C, thiamin, riboflavin, niacin, calcium and iron both at breakfast and throughout the day than non-RTEC consumers (*p *< 0.001). Mean intakes of vitamin A and phosphorus were significantly higher in non-RTEC consumers at breakfast (*p *< 0.01) but there was no difference in daily intakes. RTEC consumers had higher intake of sugar daily and at breakfast than non-RTEC consumers (Table 4).

**Table 4.** Comparison of nutrient intakes between RTEC and non-RTEC consumers (*n *= 1819).

Nutrient	RTEC consumers (*n *= 331)			Non-RTEC consumers (*n *= 1488)			*p*-value^†,a^	*p*-value^†,b^
	Breakfast intake (Mean±SD)	Daily intake (Mean±SD)	Breakfast intake as a % of daily intake (%)	Breakfast intake (Mean±SD)	Daily intake (Mean±SD)	Breakfast intake as a % of daily intake (%)		
Energy, kcal	441 ± 186	1816 ± 314	24.5	466 ± 197	1794 ± 312	26.3	0.502	0.063
Protein, g	14.9 ± 7.9	81.1 ± 26.1	19.3	15.7 ± 8.2	79.8 ± 23.8	20.8	0.696	0.746
% Energy from protein	13.5 ± 3.8	17.9 ± 4.7		13.4 ± 4.1	17.8 ± 4.3		0.916	0.768
Carbohydrate, g	61.8 ± 23.2	228.0 ± 49.1	27.6	62.3 ± 25.4	220.5 ± 47.6	28.8	0.005	0.005
% Energy from carbohydrate	57.7 ± 10.0	50.3 ± 7.3		55.1 ± 12.0	49.2 ± 7.4		<0.001	0.011
Fat, g	14.8 ± 8.1	68.2 ± 17.6	22.4	17.8 ± 9.5	68.7 ± 17.7	26.5	<0.001	0.017
% Energy from fat	29.6 ± 8.7	33.7 ± 5.7		33.6 ± 10.6	34.4 ± 5.8		<0.001	0.048
Total Sugar, g^‡^	17.1 ± 15.6	41.7 ± 21.8	47.1	9.5 ± 8.8	32.5 ± 20.9	34.2	≤0.001	≤0.001
Vitamin A, mg RE	156.9 ± 161.5	1026.2 ± 432.6	16.9	222.7 ± 219.7	1011.7 ± 441.8	23.3	<0.001	0.429
Vitamin C, mg	21.2 ± 11.5	72.6 ± 43.7	36.7	10.4 ± 9.1	54.2 ± 39.8	25.0	<0.001	<0.001
Thiamin, mg	0.6 ± 0.3	1.2 ± 0.5	50.2	0.3 ± 0.2	0.9 ± 0.4	38.1	<0.001	<0.001
Riboflavin, mg	0.8 ± 0.4	1.7 ± 0.6	48.0	0.4 ± 0.2	1.3 ± 0.5	34.2	<0.001	<0.001
Niacin, mg	6.7 ± 3.3	16.2 ± 4.9	41.9	4.0 ± 2.4	13.4 ± 4.7	30.6	<0.001	<0.001
Sodium, mg	390.5 ± 305.2	1901.1 ± 734.5	21.3	573.7 ± 395.9	2037.8 ± 751.9	29.1	<0.001	<0.001
Calcium, mg	315.0 ± 159.1	741.9 ± 254.4	43.4	192.5 ± 109.2	586.2 ± 225.2	34.1	<0.001	<0.001
Iron, mg	6.4 ± 3.1	20.1 ± 6.6	33.3	6.2 ± 4.7	19.2 ± 7.3	32.6	0.007	0.013
Phosphorus, mg	214.6 ± 156.8	1239.6 ± 453.5	18.6	251.6 ± 158.1	1240.8 ± 440.6	21.9	0.002	0.527

^†^Data were adjusted for region, area, age (years), ethnic group, income group, father’s education level, mother’s education level and energy intake (except for the analysis for energy) using ANCOVA. *P *< 0.05 was considered statistically significant. ^‡^Data were incomplete as some of the foods and beverages do not have information on the sugar content. ^a^Intake at breakfast between RTEC consumers and other breakfast consumers. ^b^Daily intake between RTEC consumers and other breakfast consumers.

**Figure 1.**
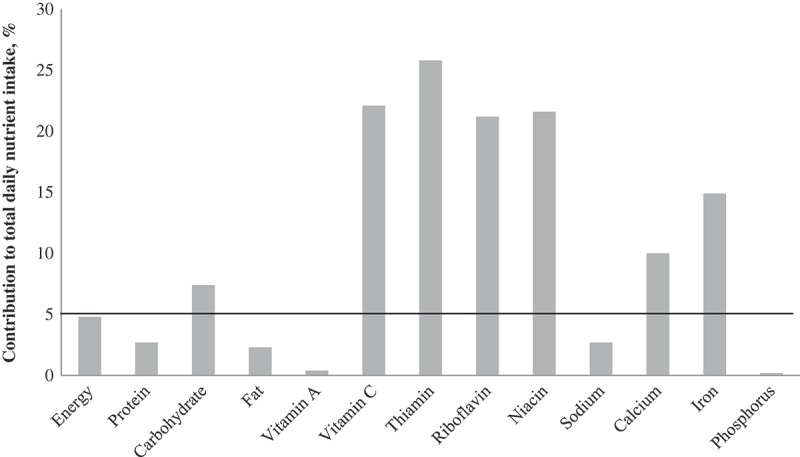
Percentage contribution of RTEC to total daily nutrient intake among RTEC consumers (*n *= 347).

Breakfast contributed to a similar proportion of daily energy intakes in both RTEC (24.5%) and non-RTEC (26.3%) consumers. Non-RTEC consumers had a higher percentage of daily fat from breakfast (26.5%) than RTEC consumers (22.4%). Breakfast contributed to 30–50% of daily intakes of vitamin C, riboflavin, niacin, calcium and iron in RTEC consumers whereas it contributed 25–38% to intakes of these nutrients in non-RTEC consumers. Among RTEC consumers, RTEC alone contributed only 5% to daily energy intakes but contributed up to 25.8% of the daily intake for thiamin, 22.1% for vitamin C, 21.6% for niacin, 21.2% for riboflavin, 14.9% for iron and 10.0% for calcium (Figure 1).

The prevalence of children not meeting at least 80% of the RNI for vitamin C, thiamin, riboflavin, niacin and calcium were significantly higher among non-RTEC than RTEC consumers, and in both types of consumers the prevalence was higher among children in the older age group (10–12 years) than children in the younger age group (6–9 years) (Table 5). Notably approximately two thirds of all children did not meet the at least 80% of the RNI for calcium. Among 6–9-year-old boys and girls, 45.3% and 55.2% of non-RTEC consumers did not achieve at least 80% of the RNI for calcium respectively, while the prevalence was 18.9% and 27.7% among male and female RTEC consumers respectively. In the older children (10–12 years) 80% of boys and 87.5% of girls who were non consumers of RTEC did not achieve at least 80% of the RNI while the corresponding percentages in RTEC consumers was 54.% and 70% respectively.

**Table 5.** Distribution of RNI achievements among RTEC and non-RTEC consumers according to gender.

	Boys					Girls				
	**RNI**^†^	RTEC consumers	Non-RTEC consumers			RNI	RTEC consumers	Non-RTEC consumers		
		RNI achievement, *n* (%)	RNI achievement, *n* (%)				RNI achievement, *n* (%)	RNI achievement, *n* (%)		
Variables		<80	<80	*χ*^2^	*p*-value		<80	<80	*χ*^2^	*p*-value
Vitamin A, mg RE										
6–9 years	500	1 (1.1)	29 (7.3)	4.87	0.027	500	13 (9.2)	34 (6.3)	1.53	0.216
10–12 years	600	3 (6.5)	32 (11.6)	1.05	0.306	600	5 (7.1)	50 (12.8)	1.78	0.182
Vitamin C, mg										
6–9 years	35	11 (12.2)	109 (27.5)	9.17	0.002	35	12 (8.5)	162 (29.8)	26.83	<0.001
10–12 years	65	15 (32.6)	157 (56.9)	9.34	0.002	65	32 (45.7)	226 (57.7)	3.43	0.064
Thiamin, mg										
6–9 years old	0.9	10 (11.1)	131 (33.0)	17.09	<0.001	0.9	14 (9.9)	217 (40.0)	45.14	<0.001
10–12 years	1.2	8 (17.4)	166 (60.1)	29.02	<0.001	1.1	24 (34.3)	224 (42.9)	12.48	<0.001
Riboflavin, mg										
6–9 years	0.9	0 (0.0)	19 (4.8)	4.48	0.034	0.9	0 (0.0)	40 (7.4)	11.03	<0.001
10–12 years	1.3	3 (6.5)	91 (33.0)	13.34	<0.001	1.0	5 (7.1)	60 (15.3)	3.27	0.070
Niacin, mg										
6–9 years	12	3 (3.3)	78 (19.6)	14.08	<0.001	12	8 (5.7)	130 (23.9)	23.19	<0.001
10–12 years	16	5 (10.9)	102 (37.0)	12.09	0.001	16	22 (31.4)	188 (48.0)	6.55	0.011
Calcium, mg										
6–9 years	700	17 (18.9)	180 (45.3)	21.31	<0.001	700	39 (27.7)	300 (55.2)	34.08	<0.001
10–12 years	1000	25 (54.3)	221 (80.1)	14.47	<0.001	1000	49 (70.0)	343 (87.5)	14.15	<0.001
Iron, mg										
6–9 years	6	0 (0.0)	3 (0.8)	0.68	0.408	6	0 (0.0)	2 (0.4)	0.52	0.470
10–12 years	10	0 (0.0)	3 (1.1)	0.51	0.477	9	0 (0.0)	5 (1.3)	0.90	0.342

^†^RNI – Recommended Nutrient Intake (NCCFN, 2005)

## Discussion

This is the first nationwide study on the comparison of breakfast choice, specifically between an RTEC and non-RTEC based breakfast among primary school children in Malaysia. Overall, 18% of children consumed an RTEC at breakfast, the prevalence of which was positively associated with a younger age, Indian ethnicity, urban area of residence, and higher income and education level of parents. Compared to non-RTEC consumers, RTEC consumers had higher intakes of several important nutrients at breakfast including calcium, iron, vitamin C, thiamin, riboflavin and niacin and higher daily intakes and adequacy of many of these nutrients.

The association of RTEC consumption in Malaysia with urban areas, income and education level is likely due to several interrelated factors including the greater availability of RTEC in urban areas and the generally higher levels of income and education among urban dwellers compared to rural dwellers. The mean household income of Malaysians in urban and rural areas was MYR6833 and MYR3831 (USD1748 and USD980) per month respectively in 2014.[19] Furthermore, the modernization and nutrition transition that occur more rapidly in the urban areas might have slowly changed the dietary habits of children from consumption of traditional breakfast foods that require cooking to ready-to-eat foods that require minimal preparation. In a study which investigated breakfast consumption, specifically RTEC consumption in an urban sample of 382 children aged 10–11 years from Kuala Lumpur, it was found that as many as 93% of children reported consumption of RTEC at least once per week and almost half consumed them two to three times per week.[20] In this mainly middle income urban sample, the higher cost of RTEC did not appear to be a barrier to consumption. Among the most cited reasons by parents for purchasing RTECs were health, taste, enhanced satiety and short preparation time.[20]

The findings that children who consume RTEC at breakfast have higher intakes of several nutrients throughout the day than those not consuming RTEC is in agreement with several other studies from around the world. A recent review on the benefits of breakfast cereal consumption revealed that out of 30 cross sectional studies conducted in seven countries (USA, Australia, UK, France, Greece, Spain and Iceland), the majority reported a higher daily intake of carbohydrate (as a percentage of daily energy), fibre, total sugars, vitamin A, vitamins B1, B2, B3, B6 and folate, vitamin D, calcium, iron, magnesium and zinc in children regularly consuming RTEC compared to those consuming them less frequently or not at all.[4] The majority of these studies also reported no differences in daily energy intake, percentage energy from protein and a lower percentage energy from fat in RTEC consumers compared to non-consumers, which concurs with our findings. Furthermore, in line with our results micronutrient adequacy tended to be higher in RTEC consumers compared to non-consumers in several of these studies, especially for calcium.[21–26] Of note, the lowest level of inadequate intake in the current study was for calcium, with about two thirds of all children failing to meet 80% of the RNI, and this was more prevalent in the older children. The nationwide SEANUTS study also recently highlighted calcium as a nutrient of concern in Malaysian children.[27] In the current study, the percentage of children achieving at least 80% of the RNI for calcium was twice as high in RTEC consumers aged 6–9 years and around 1.5 times higher in RTEC consumers aged 10–12 years compared to non-consumers. Higher consumption of milk in RTEC consumers is a probable factor explaining the higher calcium intakes as 56% and 25% of RTEC consumers reporting consumption of UHT and powdered milk respectively compared to only 8.6% and 12.2% respectively.

Similar to our findings, another study on Malaysian urban children also reported higher daily intakes of the B vitamins and vitamin C in RTEC consumers.[20] The reasons for higher intakes of these mironutrients in RTEC consumers is likely related to the fortification of these cereals. RTEC alone made a significant contribution to daily micronutrient intakes, providing 10% to calcium intakes, 15% to iron intakes, and more than 20% to intakes of vitamin C, thiamin, riboflavin and niacin, which probably reflects the fortification profile of RTECs in the Malaysian market. Intake of phosphorus, however, was lower among RTEC consumers than non-consumers, which may be because phosphorus is naturally higher in foods such as eggs and meat that were consumed more often by non-RTEC consumers at breakfast. No differences in the daily intakes of phosphorus were noted, suggesting that other meals in the day compensated for the lower intake at breakfast among RTEC consumers. Intake of vitamin A was also lower among RTEC consumers than non-consumers at breakfast. This may be because only some RTEC were fortified with vitamin A, others were not, and vitamin A is also naturally present in eggs and meat which were consumed more often by non-RTEC consumers than RTEC consumers at breakfast. However, there was also no significant difference in the daily intake of vitamin A among RTEC consumers and non-consumers.

The contribution of RTEC to whole grain intake is another possible factor contributing to higher micro-nutrient intakes in RTEC consumers. Whole grains retain the three naturally present components of the grain (germ, endosprem and bran) and are therefore generally higher in fibre, vitamins (vitamin E, B vitamins), minerals (iron, copper, zinc, magnesium) and phytochemicals than their refined grain counterparts.[28] A previous analysis of data from the MyBreakfast study showed that RTECs contributed up to 70% of total whole grain intake in children’s diets.[9] It was also shown that foods such as bread, rice and noodles which had a high reported prevalence of consumption at breakfast in the current study amongst non-RTEC consumers contributed less than 2% to total whole grain intakes.[9] This indicates that almost all of these cereal foods are consumed in their refined version, which possibly will have a negative impact on fibre and some micronutrient intakes in this population group. However, as the Malaysian food composition tables do not contain dietary fibre we were unable estimate intakes in this study. Lastly, it should be noted that RTEC consumers may have a more nutrient dense food choice throughout the rest of day which could account for some of the differences in nutrient intakes observed.

In addition to the low consumption of wholegrain foods at breakafast, food choices in the non-RTEC consumers tended to be energy dense (e.g. *nasi lemak* and fried rice were consumed by 24% and 22% respetively). Other studies in Malaysia have reported the consumption of high fat or high sugar, energy dense foods in preschoolers and adolescents at breakfast including fried rice and noodle dishes [5] and fried traditional cakes as well as biscuits.[6] These food choices are likely to have contributed to the higher fat intakes at breakfast that we observed in non-RTEC consumers and the higher overall contribution of breakfast to daily fat intake compared to RTEC consumers (22% versus 27%). There was also a higher frequency of consumption in foods high in sugar at breakfast including condensed milk and sugar and preserves, however we were unable to accurately measure intakes of total sugars in this sample as the Malaysian food composition tables do not currently include this information. Sugar content can only be obtained from manufacturers’ labelling and in Malaysia it is not mandatory to label sugar. Therefore some products may include sugar content in their nutrition information panel, while some do not, and because of the absence of information on sugar content in the Malaysian food composition tables, the sugar content of non-packaged foods could not be obtained. Notwithstanding these limitations, the estimated total sugar intake appeared higher in consumers of RTEC than non-consumers. From the available brand information reported by participants, it was estimated that the sugar content of RTEC ranged from 15% in cornflakes to 25% in wheat based cereals. Previous studies have reported higher total sugar intakes in RTEC consumers versus non-consumers [4] but this may be in part be explained by the higher dairy intake associated with RTEC consumption. For example, in the HELENA study of European adolescents, RTEC consumers had higher intakes of total simple sugars at breakfast but with a higher proportion of lactose than sucrose.[29] While RTECs can contribute to increased micronutrient intake, efforts must be made to ensure that the sugar content is kept low. While there is a need to maintain consumer palatability of such cereals, efforts can be made to have gradual reduction of sugar over time. Futhermore, it is important that efforts are made in the future to complete the Malaysian food composition tables so that intakes and sources of sugars can be assessed and monitored.

One of the major strengths of this study is the large sample size and the nationwide sampling undertaken. Another strength is that dietary intake data were collected at brand level so that RTEC and other fortified foods in the Malaysain food composition tables could be updated for micronutrient content based on nutrition panels of packaging. The high number of unacceptable energy reporters however (60% of the sample) is a limitation of the study as it may have introduced bias into the final sample. For example, the prevalence of unacceptable energy reporters was higher among children aged 10–12 years (63.2%) than children aged 6–9 years (58.2%) and among Chinese children (68.9%) than Malay children (58.2%). Removal of unacceptable reporters limits the generalizibility of the results to the total populationas as there were more young children and less Chinese children in the sample.

We chose to remove unacceptable reporters to get a more accurate assessment of nutrient intake adequacy as their inclusion could have resulted in an over- or under-estimation of nutrient intakes and prevalences of micronutrient inadequacies. As alluded to earlier, another limitation was the lack of nutrients available in the Malaysian food composition tables, especially for nutrients of public health relevance including sugar, fatty acid composition, dietary fibre and vitamin D. Fluctuations of food intakes from day to day may lead to misclassification of participants according to RTEC consumption We estimated average intake over two days of 24-h dietary recalls or records and did not apply a statistical adjustment for usual intakes. Different nutritional assessment methods were used between age groups, though the study attempted to orient the respondent to recall/record the food accurately. Nevertheless, under- and over-reporting occurred.

## Conclusion

This study demonstrates that fortified RTEC are an important source of nutrients (mainly calcium, B vitamins and vitamin C) at breakfast and for the total daily diet in Malaysian children and their inclusion in the diet may help children to meet the recommended targets for normal growth and development. However total sugar intakes appeared to be higher in RTEC consumers. Thus, parents should be encouraged to choose varieties with a lower sugar content. Further work is needed to accurately assess the impact of RTEC consumption on daily sugar intakes and other nutrients of concern including dietary fibre. In addition, future research should attempt to better characterize different types of breakfast foods and the combinations in which they are eaten using dietary pattern analysis and relate these patterns to nutrient and health outcomes.
